# Odontogenic Keratocyst Presenting in an Elderly Male

**DOI:** 10.7759/cureus.46963

**Published:** 2023-10-13

**Authors:** Samruddhi Rathi, Vidya Lohe, Mihika Deshpande, Anvika Deshpande

**Affiliations:** 1 Oral Medicine and Radiology, Sharad Pawar Dental College, Datta Meghe Institute of Higher Education and Research, Deemed to be University, Wardha, IND

**Keywords:** inflammation, swelling, ameloblastoma, enucleation, odontogenic keratocyst

## Abstract

Keratocystic odontogenic tumor (KCOT) is a benign intraosseous cyst of the jaw. The diagnosis may be confirmed by the clinical finding, or histopathological report. Finally, treatment consists of surgical excision, and follow-up is characterized by a minor recurrence. The authors report a case of KCOT of the right mandibular region and review the various diagnoses, therapeutics, and follow-up aspects of this type of tumor. The following case report describes a case of a 55-year-old male patient with odontogenic keratocyst. It can show variable clinical-radiographic features and hence should be in differential diagnosis of intra-osseous oral lesions in old age. In elderly patients, because of physical disability, selection of treatment procedures and long-term follow-up after surgery is vital.

## Introduction

The term “odontogenic keratocyst” (OKC) was first described by Philipsen in 1956. Pindborg and Hansen in 1963 described the essential features of this type of cyst [1]. The World Health Organization Classification of Head and neck tumors (fourth edition, 2017) has revised the classification of the keratocystic odontogenic tumor (KCOT) as OKC. Therefore, the OKCs are considered now benign cysts of odontogenic origin. Odontogenic lesions are caused by odontogenesis abnormalities or odontogenic apparatus cell remains. They are a common source of odontogenic lesions in the jawbones [2]. It accounts for about 10% of all odontogenic cysts. The OKC is a type of developing odontogenic cyst that forms from the dental lamina and affects the maxillofacial region. The OKC is unique among jaw cysts as it shows a tendency for recurrence and aggressive clinical behavior. OKC has a recurrence rate of 25%-30% [3]. It is most usually observed in the mandibular posterior portion. Inflammatory mandibular dental cysts are a persistent group of osteolytic lesions emerging from the odontogenic epithelium with unusual features [4]. However, it is also prevalent in the maxilla, particularly in the canine area. This benign condition has the tendency to transform malignant into squamous cell carcinoma [5].

## Case presentation

**Figure 1:**
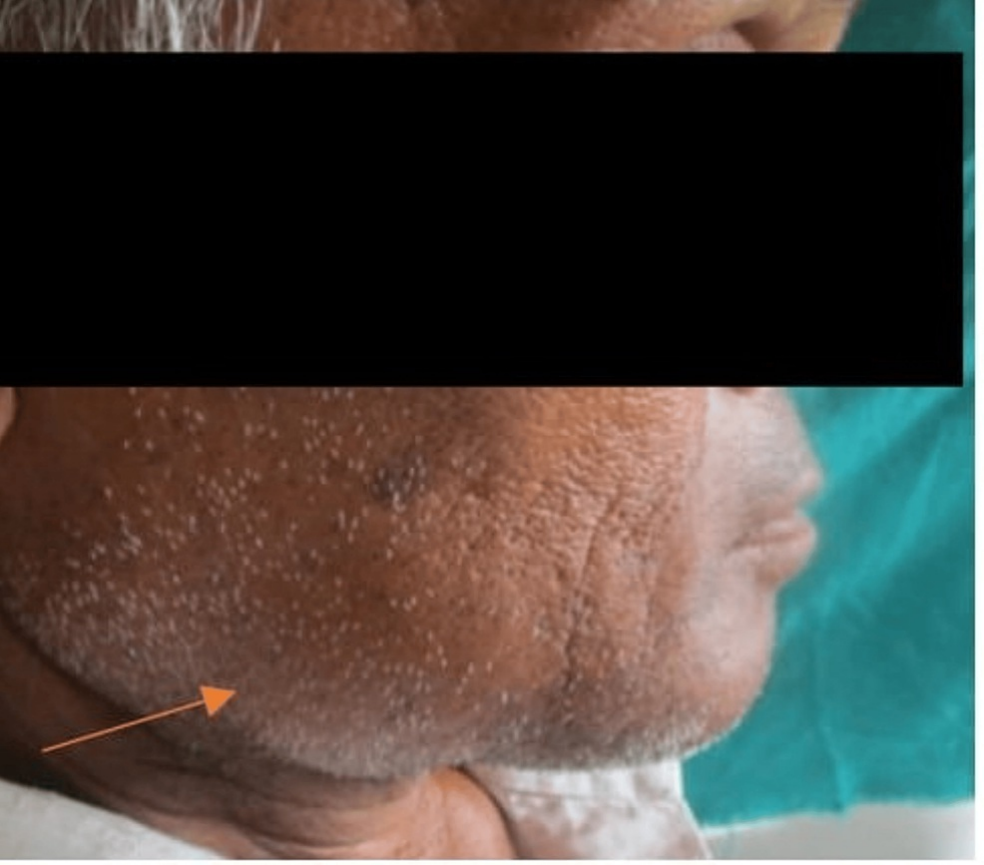
Extraoral view showing diffuse swelling over right side of face.

A 55-year-old male patient reported to an outpatient department of Oral Medicine and Radiology, with the chief complaint of swelling in the lower right posterior region of the jaw for one year and pain in the same region. The patient was apparently right one year back after which he experienced a swelling in the lower right posterior region which slowly progressed and reached to present size causing asymmetry on the right side of the face. It was associated with pain for one month. The pain was mild dull aching and intermittent in nature. The patient gave a history of mobility of the tooth in the same region for one month. No previous history of a contagious sickness or paresthesia existed. There was no sign of any negative behaviors like drinking alcohol, eating betel nuts, or smoking. The extraoral examination revealed a bilaterally asymmetrical face due to diffuse swelling on the right side of the face involving the parasymphseal region of size 5 x 3 cm approximately (Figure 1).

**Figure 2:**
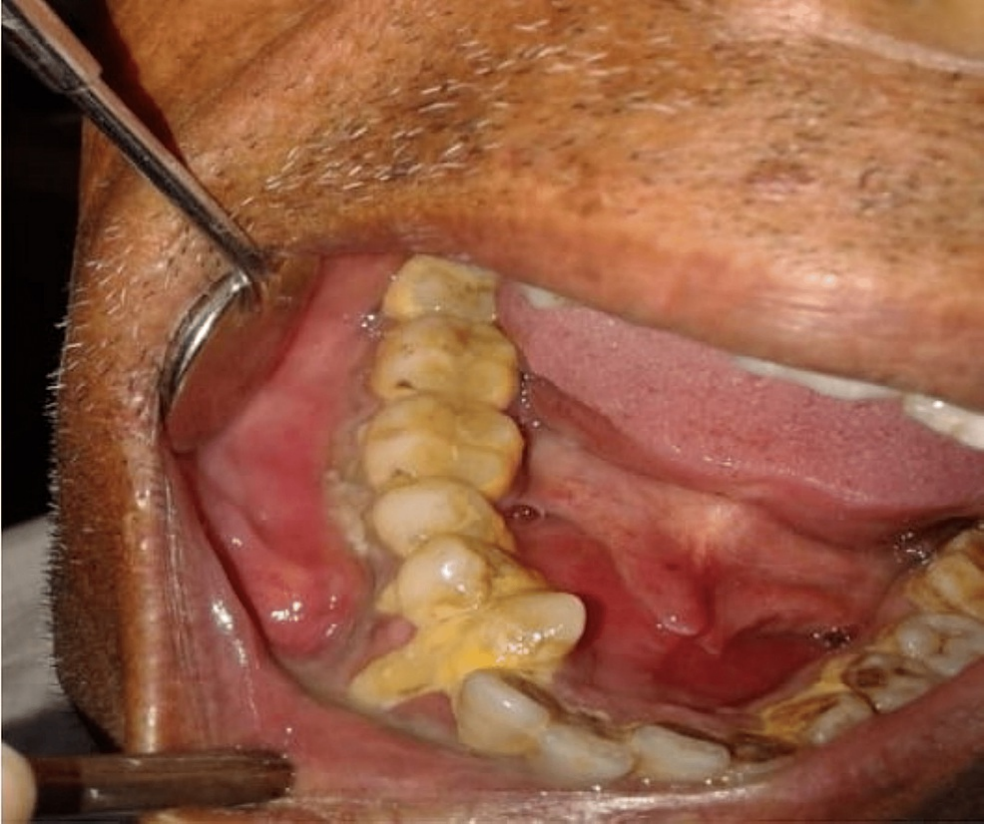
Intraoral view showing diffuse swelling in lower right posterior region of jaw with displacement of 43.

The consistency was firm, the swelling was not painful, the form was about oval, and the edges were dispersed by palpation. The local temperature was not elevated. It had a firm consistency, did not discharge, and was not sensitive. The right single submandibular lymph node was palpable at size 1 x 1 cm approximately which was round in shape, mobile, non-tender, and soft in consistency. The intraoral examination revealed a single large diffuse swelling in the lower right posterior region of the jaw (Figure 2).

Extending anteroposteriorly from mesial of 42 to distal of 47, supero-inferiorly from gingival margin till the depth of buccal and lingual vestibule of size 5 x 2 cm approximately. The shape was roughly oval, the margins were diffused and the consistency was firm. There was the expansion of buccal and lingual cortical plates in 43,44,45 regions leading to obliteration of vestibular space, causing displacement of 43, and grade I mobility with 44,45. Stains and calculus were present. As there was the presence of buccal and lingual expansion and looking at the age of the patient and location, the provisional diagnosis of ameloblastoma was made and odontogenic keratocyst was kept in the differential diagnosis.

**Figure 3:**
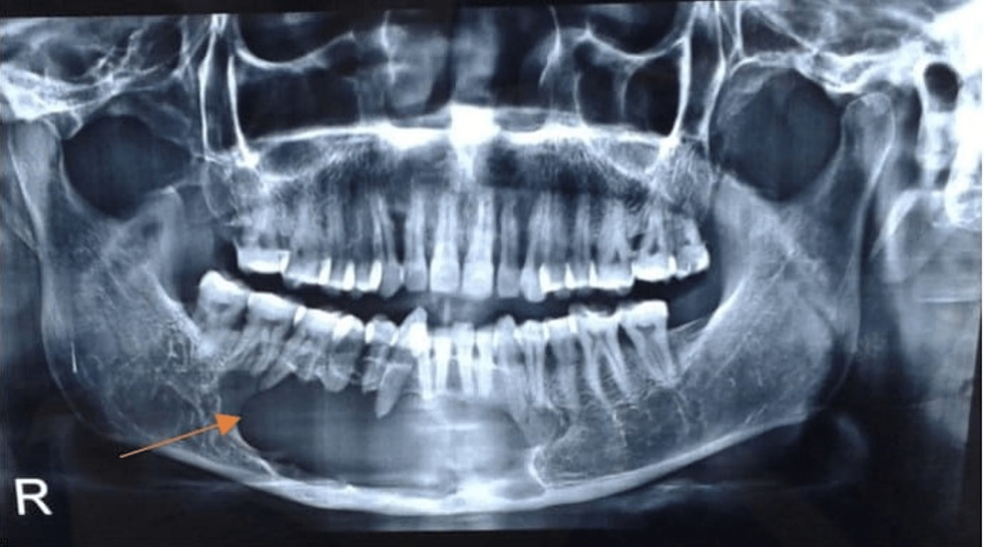
Panoramic radiograph showing a single large unilocular, well-defined radiolucency bounded by corticated margins crossing the midline with scalloped superior margins.

For the overall screening, to monitor and assess the health of your teeth and gums, a panoramic radiograph was taken which revealed a single large unilocular, well-defined radiolucency bounded by corticated margins crossing the midline with scalloped superior margins extending from 47 to 35, supero-inferiorly extending from alveolar crest to inferior border of the mandible in 47 to 35 region keeping the border intact (Figure 3).

**Figure 4:**
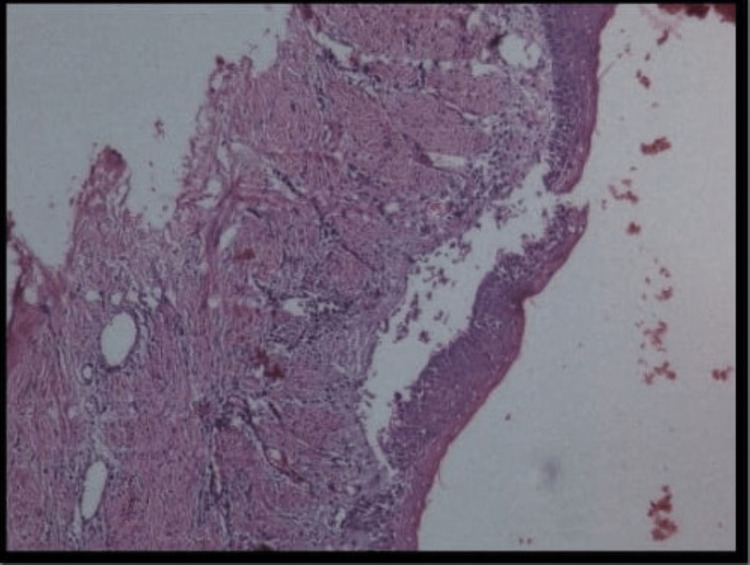
Histopathologic view showing atrophic epithelium with underlying connective tissue shows chronic inflammatory infiltrate.

According to the above radiographic findings, a provisional diagnosis of odontogenic keratocyst was made and ameloblastoma was kept in the differential diagnosis. Following this aspirational biopsy was advised and the histopathological evaluation confirmed the diagnosis of odontogenic keratocyst (Figure 4).

The lesion was surgically treated that is enucleation and the specimen was sent for histopathological evaluation and long-term follow-up was ensured.

## Discussion

OKC mostly expands in the anteroposterior dimension, this lesion may attain a huge size without causing much deformation in the jaw skeleton [6]. This case report of a 55-year-old male presented in a rare age range but at a common location of the mandibular right posterior region of the jaw. There is a noticeable gap in the recorded chronological range of OKCs, with prevalence spiking in the later years of lifespan and a little male preponderance. Rapid growth is caused by increased activity of the cyst lining's epithelial cells, which stimulates the prostaglandins' osteolytic action in the cystic lining's cell population, as well as increased accumulation of hyperkeratotic scales in the cyst lumen, resulting in a greater difference in hydrostatic pressure. Therapeutic marsupialization, enucleation, Carnoy's cure, and marginal or extreme excision are some of the treatments that can be used to treat KCOT. OKCs/KCOTs have a significant tendency for postoperative recurrence (30%-60%). Missing portions of the crater barrier, dental lamina remnants, and the emergence of daughter cysts or satellite cysts are also causes of high recurrence rates. Because recurrence of this lesion can be delayed for a long time, each case of OKC should be followed up on with yearly radiographs for at least five years following surgery [6].

As there was the expansion of buccal and lingual cortical plates in 43,44,45 region leading to obliteration of vestibular space, and looking at the age of the patient and site of the lesion, the provisional diagnosis of ameloblastoma was made and odontogenic keratocyst was kept in the differential diagnosis. In most cases, multilocular lesions favoring the mandibular posterior region showing expansion is considered as ameloblastoma and its variants depending on the age and its presenting characteristics as ameloblastoma whereas unilocular lesions are labeled as odontogenic cyst both clinically and radiographically. Regardless of the type of treatment, a patient must always be followed up with over an extended period of time [7-9]. A detailed examination of cystic fluid which is pale in color and contains keratotic squames. The protein content of cyst fluid below 4g% components could reveal important information about the probable expansion of odontogenic cysts [10]. The swelling in this case was mild as compared to the bone destruction actually present this might be the reason that it took a long time to seek any consultation. When the patients report late conservative treatment is not indicated which leads to considerable morbidity The patient did not experience any swelling or pain but as the lesion advanced it led to pain and swelling. This is because when the OKC involves the body of the mandible, it grows in the anteroposterior direction along the medullary portion of bone and hence shows minimal expansion [11].

Even though a single OKC is found in the present case, as a routine practice thorough screening of the entire jaw was carried out as it is mandatory to exclude the presence of additional OKCs. This is because Multiple OKCs that have occurred over the patients' lifetimes may be a sign of Gorlin-Goltz syndrome. Therefore, a complete evaluation is necessary in such cases [12]. There are two approaches to treating keratocystic odontogenic tumors: 1) A cautious approach and 2) an assertive approach.

Conservative treatment options should always be explored initially in young patients (children) because vigorous therapy might have negative consequences on the growth of the affected jaw, tooth formation, and the eruption process. Furthermore, marsupialization followed by enucleation was reported to have the lowest recurrence rate when compared to other conservative treatment techniques. Pogrel defines marsupialization as the process of converting a cyst into a pouch. Following that, the lesion will be decompressed [13]. Although the patient presented at a late age and there was the presence of expansion of buccal and lingual cortical plates, the resorption of roots of the teeth in the vicinity of the lesion, a characteristic feature of ameloblastoma, was not present in this case [14]. The treatment modality varies from region to region. Huge lesions, especially the parakeratotic types, are dealt aggressively with enucleation and resection [15].

## Conclusions

In conclusion, the OKC is a type of developing odontogenic cyst that forms from the dental lamina and affects the maxillofacial region. The best way to diagnose KCOTs may be to combine accurate clinical, radiographic, and trans-surgical observations with a biopsy specimen examination; this approach will help determine the most effective treatment, thereby preventing recurrences. A thorough clinical examination, patient education regarding the condition he is suffering from, appropriate investigations, adequate treatment, and meticulous follow-up to avoid recurrence is the key to the successful management of odontogenic keratocyst.
